# Complete Genome Sequence of *Methylobacterium aquaticum* Strain 22A, Isolated from *Racomitrium japonicum* Moss

**DOI:** 10.1128/genomeA.00266-15

**Published:** 2015-04-09

**Authors:** Akio Tani, Yoshitoshi Ogura, Tetsuya Hayashi, Kazuhide Kimbara

**Affiliations:** a Institute of Plant Science and Resources, Okayama University, Okayama, Japan; b Department of Genomics and Bioenvironmental Science, Division of Microbial Genomics, Frontier Science Research Centre, University of Miyazaki, Miyazaki, Japan; c Department of Infectious Diseases, Faculty of Medicine, Division of Microbiology, University of Miyazaki, Miyazaki, Japan

## Abstract

*Methylobacterium* species colonize plant surfaces and utilize methanol emitted from plants. *Methylobacterium aquaticum* strain 22A was isolated from a hydroponic culture of a moss, *Racomitrium japonicum*, and is a potent plant growth promoter. The complete genome sequencing of the strain confirmed the presence of genes related to plant growth promotion and methylotrophy.

## GENOME ANNOUNCEMENT

*Methylobacterium* species are known as one of the predominant bacterial species that inhabit plant surfaces (1). Plants emit methanol as a product of pectin demethylation (2). The methylotrophic characteristics of the genus confer an advantage for survival in the phyllosphere (3). Some species have been reported to be capable of promoting plant growth (4, 5). *Methylobacterium aquaticum* strain 22A was isolated from a hydroponic culture sample of *Racomitrium japonicum* and shown to be a potent growth promoter for various plants (5). The strain belongs to a new lineage of the genus *Methylobacterium*, because its 16S rRNA gene sequence showed low homology with that of its closest type strain, *M. aquaticum* (98.506%) (6). The genome of strain 22A was completely sequenced in this work.

Genome sequencing of the fragment library (size, 900 bp; total, 146 million bp; 316,194 reads) and paired-end library (size, 8 kbp; total, 69 million bp; 156,789 reads) was performed using the FLX 454 genome sequencer. Additional sequencing data were obtained with an Illumina 500-bp paired-end library (117 million bp, 816,704 reads). Assembly was done with Newbler version 3.0, resulting in 9 scaffolds with 134 contigs at 47× coverage. Gaps were closed *in silico* using GenoFinisher (7) and by sequencing of the PCR products bridging the scaffolds and contigs. Finally, the total genome size was found to be 7,557,960 bp (G+C content, 69.1%), with a circular chromosome (5,348,274 bp; G+C content, 71.1%) and five circular plasmids (i.e., pMaq22A-1, 1,571,989 bp [70.9% G+C content]; pMaq22A-2, 462,889 bp [67.5% G+C content]; pMaq22A-3, 85,702 bp [67.8% G+C content]; pMaq22A-4, 50,170 bp [70.3% G+C content]; and pMaq22A-5, 38,936 bp [66.72% G+C content]).

The identification of protein-coding sequences (CDSs) and annotation were carried out using the NCBI Prokaryotic Genome Annotation Pipeline (PGAP) (http://www.ncbi.nlm.nih.gov/genome/annotation_prok/) and the Microbial Genome Annotation Pipeline (MiGAP) (http://www.migap.org). The CDSs that were inconsistent between the two pipelines were manually corrected using GenomeMatcher (7). The predicted number of CDSs was 6,944. Ten *rrn* operons were found in the chromosome and one in pMaq22A-1. Eighty-three tRNA genes were found in the chromosome, and 12 were in pMaq22A-1. Thus, it may be possible to consider pMaq22A-1 the second chromosome.

We identified almost all important genes encoding proteins involved in methylotrophy (8, 9): methanol dehydrogenases (MxaFI and XoxF), methylotrophy regulatory proteins (MxcQE and MxbDM), and PQQ synthesis enzymes (PqqA1A2BCDE, *pqqA* gene is duplicated, and PqqFG are encoded in distant loci). We also identified the genes important for interaction with plants: *trans*-zeatin production (*miaA*), cobalamin synthesis (*cob*), 1-aminocyclopropane-1-carboxylate deaminase (*acdS*), siderophore production (*rhbC*), and five copies of flagellin genes (*flaLabcde*), but no nitrogenase gene. Nif genes were found in nitrogen-fixing *Methylobacterium nodulans* and *Methylobacterium populi* (10, 11), which is consistent with the fact that this strain is not diazotrophic. The functions of these genes are under investigation. The genome information of the strain will allow both further functional and comparative genome analyses among the genus *Methylobacterium* and the establishment of a novel species to which the strain belongs to as a new lineage.

### Nucleotide sequence accession numbers.

The complete genome data have been deposited in DDBJ under the accession numbers AP014704 to AP014709. The versions described in this paper are the first versions, AP014704.1 to AP014709.1.
